# 

**Published:** 1885-01

**Authors:** 


### CONTENTS.

ORIGINAL COMMUNICATIONS—
An Anomalous Tumor. By J. McF. Gaston, M. D., Atlanta, Ga. . 577
Hydrochlorate of Cocaine. By J. M. Hull, M. D., Augusta, Ga. . 586
SOCIETY REPORTS—
Macon Medical Society. . . . . . . . . . . . . . . . . . . . 588
CLINIC REPORTS—
Pelvic Abscess—A Clinical Lecture. By Prof. C. A. Lee Reed, M. D., Cincinnati. . . . . . . . . . . . . . . . . . . . 597
Phthisis Pulmonalis—A Clinical Lecture. By Prof. W. D. Bizzell, M. D., Atlanta, Ga. . . . . . . . . . . . . . . . . . 603
Hydrochlorate of Cocaine—A Clinical Lecture. By Prof. A. W. Calhoun, M. D., Atlanta, Ga. . . . . . . . . . . . . . 608
BOOK REVIEWS—
A Text-Book of Practical Medicine. By Alfred L. Loomis, M. D., LL. D., Professor of Practical Medicine in the Medical Department of the University of the City of New York. . . . . . 611
Transactions of the Medical Association of Georgia, 1884. . . 622
A Manual of Obstetrics. By Edward L. Partridge, M. D., Professor of Obstetrics, New York Post-Graduate School. . . . . . 627
A Practical Treatise on Massage—Its History—Mode of Application and Effects. By Douglas Graham, M. D., Fellow of Massachusetts Medical Society. . . . . . . . . . . . . 629
A Theoretical and Practical Treatise on the Hemorrhoidal Disease. By William Bodenhamer, A. M., M. D. . . . . . 630
A Manual of Bandaging. By C. Henri Leonord, A. M., M. D., Professor Medical and Surgical Diseases of Women, Michigan College of Medicine. . . . . . . . . . . . . . . . . . . . 631
EDITORIAL—
Medical Legislation. . . , . . . . . . . . . . . . . . . . . 633
Athletic Sports. . . . . . . . . . . . . . . . . . . . . . 634
Items. . . . . . . . . . . . . . . . . 635, 636, 637, 638, 639, 640

